# Characterisation of a novel *OPA1* splice variant resulting in cryptic splice site activation and mitochondrial dysfunction

**DOI:** 10.1038/s41431-022-01102-0

**Published:** 2022-05-09

**Authors:** Joshua Paul Harvey, Patrick Yu-Wai-Man, Michael Edward Cheetham

**Affiliations:** 1 UCL Institute of Ophthalmology, London, UK; 2 Moorfields Eye Hospital NHS Foundation Trust, London, UK; 3 Cambridge Eye Unit, Addenbrooke’s Hospital, Cambridge University Hospitals, Cambridge, UK; 4 Department of Clinical Neurosciences, John van Geest Centre for Brain Repair and MRC Mitochondrial Biology Unit, University of Cambridge, Cambridge, UK

**Keywords:** Medical genetics, Neurodegeneration, RNA splicing, Visual system

## Abstract

Autosomal dominant optic atrophy (DOA) is an inherited optic neuropathy that results in progressive, bilateral visual acuity loss and field defects. *OPA1* is the causative gene in around 60% of cases of DOA. The majority of patients have a pure ocular phenotype, but 20% have extra-ocular features (DOA +). We report on a patient with DOA + manifesting as bilateral optic atrophy, spastic paraparesis, urinary incontinence and white matter changes in the central nervous system associated with a novel heterozygous splice variant NM_015560.2(*OPA1*):c.2356-1 G > T. Further characterisation, which was performed using fibroblasts obtained from a skin biopsy, demonstrated that this variant altered mRNA splicing of the *OPA1* transcript, specifically a 21 base pair deletion at the start of exon 24, NM_015560.2(*OPA1*):p.Cys786_Lys792del. The majority of variant transcripts were shown to escape nonsense-mediated decay and modelling of the predicted protein structure suggests that the in-frame 7 amino acid deletion may affect OPA1 oligomerisation. Fibroblasts carrying the c.2356-1 G > T variant demonstrated impaired mitochondrial bioenergetics, membrane potential, increased cell death, and disrupted and fragmented mitochondrial networks in comparison to WT cells. This study suggests that the c.2356-1 G > T *OPA1* splice site variant leads to a cryptic splice site activation and may manifest in a dominant-negative manner, which could account for the patient’s severe syndromic phenotype.

## Graphical abstract

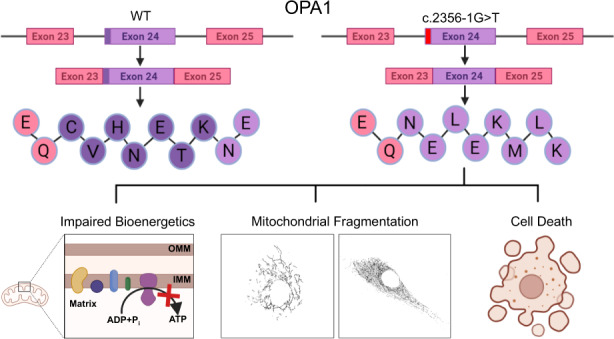

## Introduction

Autosomal dominant optic atrophy (DOA [OMIM 165500]) is the most common inherited optic neuropathy in the general population with an estimated minimum prevalence of 1 in 25,000 [1]. Patients typically present in early childhood with progressive bilateral irreversible loss of vision. However, the visual loss associated with DOA is highly heterogenous, ranging from 6/6 to detection of hand movement vision [2]. Although dysfunction is restricted to the retinal ganglion cells (RGCs) and optic nerve degeneration in most patients, ~20% of patients demonstrate extraocular features, the commonest of which are sensorineural hearing loss, peripheral neuropathy and ataxia [1, 3].

Most cases of DOA ( > 60%) are caused by variants in the *OPA1* gene, which encodes for a multimeric GTPase protein that localises to the inner mitochondrial membrane [1]. There is an expanding list of nuclear-encoded genes associated with both dominant and recessive inherited optic neuropathies, including, *WFS1*, *OPA3, SSBP1, ACO2, MFN2, SPG7, NR2F1, SLC25A46* and *DNAJC30* [4].

*OPA1* localises to 3q28-q29 and it spans over 60 kb with 30 exons and 8 isoforms produced by alternative splicing. Over 400 potential pathogenic variants of *OPA1* have been reported (https://www.lovd.nl/*OPA1*), with the majority of disease associated variants being single heterozygous variant; although compound homozygous and heterozygous variants have also been identified, often presenting earlier in life due to their more severe phenotypes [5, 6]. Homozygous *OPA1* null mutations are thought to be embryologically lethal, at least in mice [7, 8]. Pathogenic *OPA1* variants are diverse, both with respect to type and the domain affected. In a North of England cohort, variants were characterised as missense (15.4%), nonsense (15.4%), splice site (38.4%) and deletions (30.8%) [1]. Many variants in *OPA1* are thought to manifest as premature termination codons (PTCs) that result in nonsense-mediated decay (NMD), leading to reduced protein and a haploinsufficient disease mechanism [9]. However, some variants have been hypothesized to exert a dominant-negative effect on the wild-type (WT) allele [3]. The majority of these variants are thought to be missense variants with the mutant transcript likely escaping NMD and the altered protein interfering with the function of WT protein. This correlates clinically with the observation that missense variants and those involving the central GTPase domain carry a significantly higher risk of a more severe multisystemic DOA + phenotype [3]. Missense variants may give rise to proteins that escape protein folding quality control and their subsequent dominant-negative effect could enhance the loss of *OPA1* function resulting in non-RGC cell types being affected. Splice site variants, however, are classically associated with pure optic atrophy (pOA) presumably because most cause exon skipping or intron retention, leading to truncations or insertions with frame shifts, and PTCs with subsequent haploinsufficiency [10–13].

We previously reported a 60-year-old woman with a DOA + phenotype characterised by multiple sclerosis-like features who was found to carry a NM_015560.2(*OPA1*):c.2356-1 G > T splice site variant hereafter referred to as c.2356-1 G > T [14]. The patient presented with a spastic gait and increased lower limb tone and ankle clonus with brisk reflexes. Visual acuity was 6/9 in both eyes with dyschromatopsia and temporal optic disc pallor. An MRI scan showed multiple high T2 signal lesions in the brain and spinal cord and unmatched oligoclonal bands were detected in the cerebrospinal fluid. Dermal fibroblasts were established from a skin biopsy and used for in vitro modelling of this *OPA1* variant.

## Materials and methods

### In silico modelling

Splice site predictions were performed using MaxEnt (http://www.hollywood.mit.edu/), Human Splicing Finder, Genomis (http://www.umd.be/Redirect.html), Cryp-Skip (https://www.cryp-skip.img.cas.cz/), NetGene2 (https://www.services.healthtech.dtu.dk/) and Splice AI (https://www.spliceailookup.broadinstitute.org) [15–18]. Protein modelling was performed with Phyre^2^ (http://www.sbg.bio.ic.ac.uk/) [19]. Proteins were displayed using EzMol (http://www.sbg.bio.ic.ac.uk/ezmol/).

### Cell culture

The patient provided informed consent for dermal fibroblasts to be established from a skin biopsy (NRES Committee, Yorkshire and The Humber - Leeds Bradford Research Ethics Committee (REC 13/YH/0310). Fibroblasts were grown in DMEM media (Gibco, Thermo Fisher, Waltham, Massachusetts, United States) supplemented with 10% foetal bovine serum (FBS) (Gibco) with 1% non-essential amino acids (Gibco) and 1% penicillin-streptomycin (Gibco) at 37 °C and 5% CO_2_. WT fibroblast cell lines (Human Dermal Fibroblasts (HDF)) were used as controls.

### Genotype and transcript analysis

DNA extraction from fibroblasts was performed using the Wizard Genomic DNA Purification Kit (Promega, Madison, Wisconsin, United States) as per the manufacturer’s protocol, followed by sequencing to confirm the *OPA1* genotype [20]. RNA extraction (Qiagen, Hilden, Germany) and RT-PCR (Bioline, London, United Kingdom) were performed as per the manufacturer’s protocol [21, 22]. Samples were then analysed using gel electrophoresis with 4% agarose followed by Sanger sequencing for cDNA confirmation. Quantitative PCR (qPCR) with and without emetine for 4 h (100 µg/ul) was used to quantify *OPA1* transcript number. GAPDH and ACTB were used as controls (Table 1).

**Table 1:** Primers used in PCR, RT-PCR and qPCR reactions.

Gene	Exon	Forward Primer	Reverse Primer
*OPA1*	24	TCCCTGGGTTTTCTACCCTC	GGCAAAGGTCTAGGTCGGTTT
*OPA1*	22–26	ACAGCAATGGGATGCAGCTAT	CATGCGCTGTATACGCCAAA
*OPA1*	24–26	AACCACAGTCCGGAAGAACCTTGAAT	TTTGCGGTGATAGCAAGCA
*OPA1*	qPCR	CGACCCCAATTAAGGACATCC	GCGAGGCTGGTAGCCATATTT
*GAPDH*	9	CCCCACCACACTGAATCTCC	GGTACTTTATTGATGGTACATGACAAG
*ACTB*	4	CCAACCGCGAGAAGATGA	CCAGAGGCGTACAGGGATAG

### Western blotting

Cell fractions for protein blotting were obtained following differential centrifugation at 4 °C, as previously described [23]. Cell samples were lysed in radioimmunoprecipitation buffer (RIPA) consisting of 50 mM Tris (pH 8), 150 mM NaCl, 0.1% (w/v) SDS, 0.5% sodium deoxycholate and 1% NP-40. Protein was quantified using a Pierce bicinchoninic acid assay (BCA) (Thermo Fisher) and normalised according to a 2000-0 µg protein ladder. Western blotting was performed with 15 μg of total cell protein taken from mutant and control lines. The following antibodies were used: anti-*OPA1* (NovusBio, Littleton, Colorado, United States), β-tubulin (Abcam, Cambridge, United Kingdom) and LC3 (CellSignal, Danvers, Massachusetts, United States). Secondary antibodies contained HRP-conjugated goat, mouse or rabbit IgG (Thermo Fisher). To inhibit protein degradation, fibroblasts were incubated for 6 h with 100 nM bafilomycin or DMSO in fresh fibroblast media at 37 °C before protein extraction.

### Assessment of mitochondrial membrane potential

Mitochondrial membrane potential was assessed using tetramethylrhodamine ethyl ester (TMRE)(Thermo Fisher) in combination with carbonyl cyanide 4-(trifluoromethoxy) phenylhydrazone (FCCP). Cells were plated into a black-walled clear flat bottomed 96-well plate (20,000 cells per well) and cultured for X h. A depolarization control (low mitochondrial membrane potential/low TMRE signal) was created by incubating cells with FCCP at 20 µM for 10 min at room temperature. Non-TMRE stained controls were also included for both test cells and depolarization controls. Cells were then incubated for 20 min in 200–1000 nM TMRE at 37 °C and 5% CO_2_. After incubation, cell media was aspirated and replaced in 100 µl of PBS/0.2% BSA. This was aspirated and again resuspended in 100 µl of PBS/0.2% BSA. Fluorescence was then measured using an excitation filter set at 549 nm and emission at 575 nm. Fluorescence was calculated using the following formula (Test Fluorescence – Negative Control)/(FCCP fluorescence – negative FCCP fluorescence). Samples were run in quadruplicate and data presented as means.

### Cell death assessment

Cell death was assessed using an LDH Assay (Abcam). Cells were plated into a 96-well plate (20,000 cells per well) and the following day tissue media was exchanged to contain 200 mM of colbalt chloride and then incubated for 48 h. The assay was then performed as per the manufacturer’s protocol [24]. Three controls were used: (i) background control, providing a measure of absorbance from the culture media; (ii) a low control (no cell lysis); and (iii) a high control (10 µL of cell lysis solution (Triton x100) incubated in the tissue medium). Cell death = (sample absorbance – low control) / (high control – low control) x 100. Low control = normoxic environment. High control = Triton x100 lysis buffer for 10 min. Prior to the assay being conducted, the plate was centrifuged to remove any cell remnants from the tissue media and the media was transferred to a fresh 96-well plate.

### Seahorse XF-96 bioenergetics analysis

Bioenergetics assessment was performed in live fibroblasts seeded at 20,000 cells/well in 96 well plates using the Seahorse XFe96 extracellular flux analyser (Agilent, Santa Clara, California, United States) as previously described [25]. XF base media was supplemented with 1 mM pyruvate, 2 mM glutamine and 1 mM glucose pH 7.4. Cells were treated with 1.5 µM Oligomycin, 1 µM carbonyl cyanide p-triflouromethoxyphenylhydrazone (FCCP) and 0.5 µM rotenone (Aligent). Otherwise, the experiment was performed as per the manufacturer’s protocol (https://www.agilent.com/en/product/cell-analysis/how-to-run-an-assay). Subsequently, the tissue culture plate was taken for protein quantification using the aforementioned BCA assay and measured in triplicate. Data was normalised to µg of protein and was analysed using the proprietary Wave software (Agilent) and exported to Prism (GraphPad, San Diego, California, United States).

### Mitochondrial network analysis

Fibroblasts were mounted on a glass chamber slide the day before fixation. The following day cells were incubated with Mitotracker CMXRos (ThermoFisher) at a concentration of 100 nM for 30 min at 37 °C. Cells were then washed x2 with fresh warm media before being fixed with 4%PFA/Cell media for 10 min at room temperature and then washed with PBS x3 for 5 min. Cells were permeabilised with 0.1% Triton x-100 for 10 min and then washed with PBS x3 for 5 min. Cells were stained with DAPI (1:5000 in a 1:1 mix of ddH_2_O and PBS) for 2 min at room temperature. Cells were washed with PBS before mounting with a glass cover slip and imaging. Imaging was performed using a Leica Sp8 Confocal microscope using the x63 oil immersion objective. Single cells z-stacks were taken using 0.5 µm steps. Maximal z-stack projections were then produced using imaging software (FIJI). Deconvolution was performed using the Leica software. The following workflow was performed to optimise the mitochondrial network analysis tool (MiNA). Z-stack projections were colour split to isolate the red signal. Images were then cropped to ensure only single cell analysis. Images were then binarized and skeletonised before inversion and running of the MiNA analysis tool. Mitochondrial number and volume were also assessed using un-skeletonised images using the analyse particles tool.

### Protein modelling

In silico analysis was used to model the WT and NM_015560.2(*OPA1*):p.Cys786_Lys792del protein using the PHYRE Protein Fold Recognition Engine (Phyre^2^) [19]. Proteins were then displayed using the EzMol molecular display wizard.

### Statistical analysis

Statistical analysis was performed with Prism Version 8 (GraphPad). All the results have been provided as mean with standard deviation (SD) values, unless otherwise stated.

## Results

### In silico modelling

A combination of in silico methods were used to predict the effect of the c.2356-1 G > T *OPA1* variant on splicing, all of which demonstrated a high likelihood of disruption of the AG 3’ splice site (Table 2). Two prediction tools (NetGene2 and SpliceAI) predicted a new splice site at position c.2376, 21 base pairs (bp) downstream from the original splice site.

**Table 2:** Predicted effect of the c.2356-1 G > T *OPA1* variant based on in silico splice site prediction tools.

Prediction Tool	Splice Site Disruption	New 3’ splice site prediction
MaxEnt	WT; MAXENT 6.31, MM 4.51, WMM 4.11; c.2356-1 G > T; MAXENT −2.28, MM −4.09, WMM −4.49	? Not predicted
Human Splicing Finder, Genomnis	MaxEnt Acceptor: 4.65 > −3.94 (−184.73%) HSF: Consensus values 73.74 > 45.87 (−37.79%)	? Not predicted
Cryp-Skip	P_cr-E_ (52%) P_ExSK_ (48%)	Most likely new 3’ splice site (AG) at c.2482 (AGAAG^TAGAT)
NetGene2		Most likely new 3’ splice site (AG) at c.2376 (CCAAG^AATGA)
Splice AI	Acceptor Loss Δ0.98 (0–1) Acceptor gain Δ0.68 (0–1)	Pre-mRNA position: c.2356-1 Pre-mRNA position: c.2376

*MAXENT* Maximum Entropy, *MM* Markov model, *P*_*cr-E*_ Probability of cryptic splice site activation, *P*_*ExSK*_ Probability of exon skipping, *WMM* Weight Matrix Model.

### Transcript analysis

**Fig. 1:**
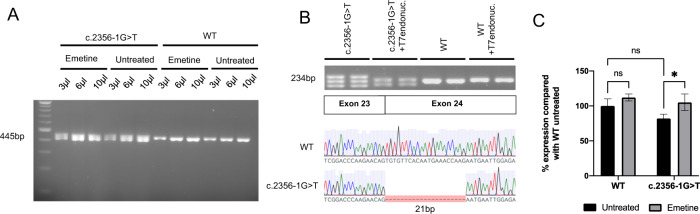
Molecular characterisation of the c2356-1G > T splice site *OPA1* variant. **A** Full length gel demonstrating an amplicon ~450 bp. No other bands suggestive of exon skipping were detected in the presence of emetine. **B** RT-PCR followed by electrophoresis revealed a separate band representing a shortened transcript in the c.2356-1 G > T cell lines. Heteroduplexes were digested with T7 endonuclease (1unit/3 µL of PCR reaction) prior to loading. Gel extraction of the lower band and Sanger sequencing demonstrated at 21 bp deletion at the start of exon 24 (lower panel). **C** qPCR quantification of *OPA1* transcript in the presence of emetine. Data represents mean ± SD of 3 biological repeats and 2 methodological repeats. Test results were normalised to GAPDH and ACTB controls. *p < 0.05. Error bars represent SD.

Genomic DNA Sanger sequencing of patient-derived fibroblasts confirmed a single heterozygous G **>** T variant at nucleotide position c.2356-1 (data not shown). The analysis of fibroblast *OPA1* transcripts by RT-PCR for a 445 bp cDNA amplicon spanning exons 22–26 (Fw:ACAGCAATGGGATGCAGCTAT; Rv:CATGCGCTGTATACGCCAAA) demonstrated an altered band pattern from the c.2356-1 G **>** T cells on a 1% agarose gel. Pre-treatment of fibroblasts with emetine to inhibit NMD of transcripts did not reveal additional gel bands (Fig. 1A). Further separation using a 4% agarose gel revealed two additional bands in the c.2356-1 G **>** T patient line separated by ~20 bp which were resolved to 2 bands of equal density after treatment with T7 endonuclease digestion prior to electrophoresis, suggesting that the third band was a heteroduplex (Fig. 1B). Sanger sequencing of the additional band revealed a cDNA product with a 21 bp deletion (c.2356_2376del) at the start of exon 24, which would result in the loss of 7 amino acids NM_015560.2(*OPA1*): p.Cys786_Lys792del hereafter referred to as p.Cys786_Lys792del (Fig. 1B). qPCR for *OPA1* transcript levels (Fw:CGACCCCAATTAAGGACATCC; Rv:GCGAGGCTGGTAGCCATATTT amplicon 102 bp) demonstrated no statistically significant reduction in the c.2356-1 G **>** T fibroblasts compared with the WT control. However, *OPA1* transcript levels were significantly increased in the c.2356-1 G **>** T fibroblasts in the presence of the NMD inhibitor, emetine (Fig. 1C).

### Protein characterisation

**Fig. 2:**
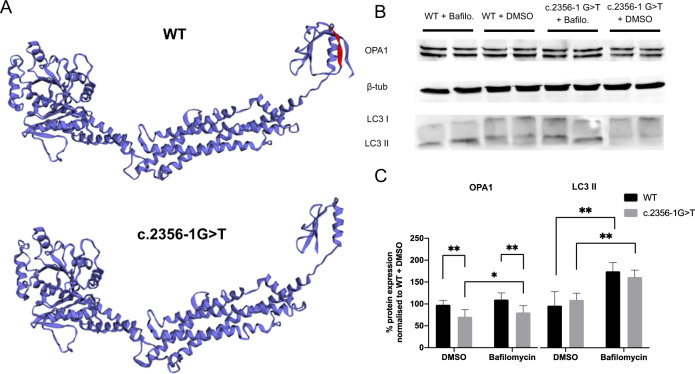
Characterisation of the mutant p.Cys786_Lys792del *OPA1* protein arising from the c.2356-1 G > T splice variant. **A** In silico 3D modelling of the OPA1 protein derived from WT and c.2356-1 G > T transcripts. Residues predicted to be skipped in c.2356-1 G > T have been highlighted in red on the WT protein. **B** Western blot of WT and c.2356-1 G > T fibroblast protein lysates after 6 h of incubation with 100 nM DMSO or 100 nM Bafilomycin at 37 °C. Proteins stained for OPA1, beta-tubulin loading control and LC3II. **C** OPA1 and LC3 densitometry normalised to loading control. Mean ± SD represent 3 biological repeats and 2 methodological repeats. *p < 0.05, **P < 0.01. Errors bars represent SD.

Modelling of the p.Cys786_Lys792del protein indicated a loss of a beta-strand and subsequent conformational change of the terminal GTPase effector domain (GED). Western blotting was suggestive of reduced, but greater than 50% *OPA1* expression in the c.2356-1 G > T fibroblasts. Although the level of expression was significantly increased after a 6-hour bafilomycin incubation, it remained lower than WT levels (Fig. 2B, C). LC3II was upregulated in keeping with successful inhibition of autophagy.

### Mitochondrial bioenergetics

**Fig. 3:**
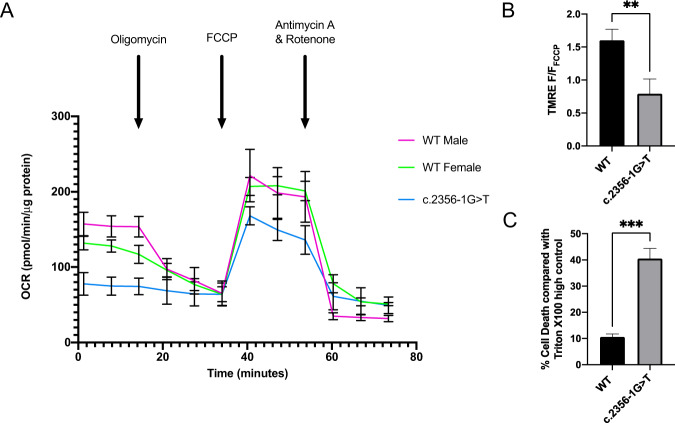
Investigation of mitochondrial bioenergetics with the Seahorse XF-96 platform. **A** Oxygen consumption rate (OCR) was calculated for the patient and control cell lines. 1 µM oligomycin, 1 µM FCCP and 0.5 µM rotenone was used. Data was exported and normalised to protein concentration using a BCA assay. *N* = 73. **B** Hypoxia-induced cell death assay. Graph represents the mean of technical triplicate values and biological quadruplicates. **C** Mitochondrial membrane potential assay. TMRE F/F_FCCP_ = (sample fluorescence – negative control) / (depolarization control – depolarization negative control). Negative control = 0 nM of TMRE. Depolarization control = 20 mM of FFCP for 10 min at RT. Graph represents the mean of technical triplicate values and biological quadruplicate. *p < 0.05, **p < 0.01, ***p < 0.005 and ****P < 0.001. Errors bars represent SD.

The impact of the c.2356-1 G **>** T variant on mitochondrial energetic output was investigated in fibroblasts with the Seahorse XF-96 platform (Fig. 3A and Table 3). c.2356-1 G **>** T fibroblasts were compared with both male and female WT control lines. Fibroblasts carrying the c.2356-1 G **>** T variant displayed significantly reduced basal respiration, maximal respiration and ATP-linked respiration compared with both control lines (Fig. 3A). Non-mitochondrial respiration was significantly higher in the c.2356-1 G **>** T line compared with the Male WT line, but not the Female WT line (Table 3). Mitochondrial membrane potential was assessed using the mitochondrial membrane sensitive dye, TMRE. The mutant *OPA1* fibroblasts had significantly reduced mitochondrial membrane potential (TMRE F/F_FCCP_) compared with controls. (Fig. 3B). Hypoxia induced with cobalt chloride treatment (200 µM for 48 hours) led to a significantly increased rate of cell death in the patient lines compared with controls (Fig. 3C).

**Table 3:** Key respiratory parameters from Seahorse XF-96 bioenergetics assay.

Bioenergetic Parameters (OCR)	WT Male	WT Female	c.2356-1 G > T	*P*-value
Basal Respiration	122 ± 12.2	64.7 ± 9.7	20.6 ± 13.7	*P* < 0.001
Maximal Respiration	171 ± 23.0	144 ± 12.3	96.0 ± 17	*P* < 0.001
ATP-linked Respiration	73.7 ± 22.9	46.7 ± 10.0	10.0 ± 14.3	*P* < 0.001
Non-mitochondrial Respiration	33.3 ± 15.0*	61.4 ± 10.2**	55.1 ± 15.7	*P* < 0.05* NS**

*P*-values represent independent comparisons between c.2356-1 G > T and both WT Male and WT Female lines unless otherwise specified with asterisk. *OCR* Oxygen consumption rate (pMol/min/µg protein). *N* = 70. Values represent mean ± SD.

*Comparison between WT Male and c.2356-1 G > T. **Comparison between WT Female and c.2356-1 G > T.

### Mitochondrial network analysis

**Fig. 4:**
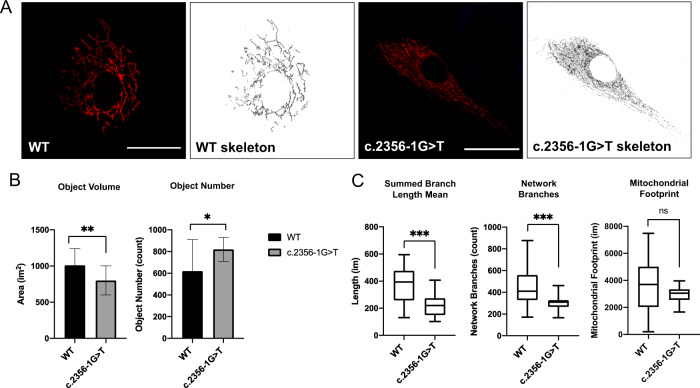
Mitochondrial network analysis. **A** Representative images maximal z-stack projections of mitochondrial networks stained with MitoTracker CMXRos. **B** Mitochondiral networks assessed after thresholding using the particle analysis tool. **C** Mitochondrial network analysis of skeletonised images using the MiNA plugin. *p < 0.05, **P < 0.01, ***p < 0.005. *N* = 20. Scale bar 25 µm.

Live fibroblasts were stained using MitoTracker CMXRos to visualise mitochondrial networks and then fixed and imaged using a confocal microscope. The mitochondrial network in c.2356-1 G > T cells were significantly more fragmented compared with WT controls (Fig. 4A). Particle analysis of maximal z-stack projections showed a significant decrease in network volume and a significant increase in object number in the c.2356-1 G > T line. Further analysis of skeletonised mitochondrial networks demonstrated a significant decrease in network branch length and the number of branches in the c.2356-1 G > T line, but no significant change in total mitochondrial mass (Fig. 4B, C).

## Discussion

Over 400 disease-causing variants in the *OPA1* gene have been reported, with considerable heterogeneity with regards to type and location [26]. Although these variants result in a wide spectrum of disease severity, several genotype/phenotype correlations have been described, in particular, the strong association between more severe visual loss and a higher likelihood of developing extraocular DOA + features with missense variants affecting the catalytic GTPase protein domain [14, 27]. The majority of splice site *OPA1* variants that have been characterised to date appear to result in exon skipping or intron retention, with resultant NMD of the variant transcripts [3, 28, 29]. Without characterisation of the transcriptional effects of splice site variants, they can be misclassified as synonymous, missense or nonsense variants, which can affect disease prognostication and counselling in the context of DOA [3, 30]. A number of in silico modelling tools are available to predict the downstream effects of *OPA1* variants on splicing [25, 31, 32]. However, in vitro studies have demonstrated a wide variability of translational and transcriptional effects of splice site variants, highlighting the limitations of in silico prediction methods [10, 33].

All 5 in silico prediction tools indicated that the c.2356-1 G > T variant was likely to disrupt the acceptor splice site at the start of exon 24. Some of the modelling also suggested cryptic splice site activation at either nucleotide 2482 or 2376, which would result in a 127 bp out-of-frame or a 21 bp in-frame deletion, respectively. In frame variants may escape NMD and could exert dominant-negative effects [34]. Since this mechanism is more closely associated with a severe DOA + phenotype, differentiating between these two possibilities is crucial to understanding the molecular pathophysiology of the c.2356-1 G > T variant [10].

Transcript analysis of patient-derived fibroblasts carrying c.2356-1 G > T revealed an in-frame 21 bp deletion at the start of exon 24, which escapes NMD resulting in a mutant protein that lacks 7 amino acid residues, p.Cys786_Lys792del (Fig. 1), as predicted by SpliceAI and NetGene2. This is an unusual consequence of splice site variants affecting *OPA1*, as similar variants that cause in-frame amino acid deletions, for example, c.1065 + 5 G > A and c.2356-8 T > G, still appear to result in haploinsufficiency [10].

In silico protein modelling of p.Cys786_Lys792del predicted the loss of a β-strand and subsequent conformational change of the GED domain, which is thought to be involved in homo-dimerization [35]. Without the introduction of a PTC, it is unlikely that NMD would be activated, in keeping with our observation of only a small effect of NMD inhibition by emetine. Nevertheless, a 7 amino acid deletion could lead to protein misfolding and activate protein quality control and degradation pathways, such as lysosomal protein degradation, which has been shown to be the primary route through which misfolded *OPA1* protein is eliminated [36, 37]. Protein blotting of c.2356-1 G > T fibroblasts showed more than 50% of WT *OPA1* levels. Furthermore, inhibition of this protein degradation pathway using bafilomycin showed only modest increases in OPA1 protein expression. Given that bafilomycin inhibits the primary mechanism through which misfolded *OPA1* is thought to be degraded, this finding suggests that the reduced c.2356-1 G > T *OPA1* protein levels could be accounted for by inter-patient variability or a small decrease in transcript number rather than significant *OPA1* degradation, and therefore suggests that some of the p.Cys786_Lys792del protein escapes degradation. Although further experimental confirmation is needed, it is possible that the p.Cys786_Lys792del protein is therefore leading to disease via a dominant-negative effect. Given the location of this deletion in the central dynamin domain, it could potentially interfere with OPA1 protein oligomerisation, which is critical to its function as a mitochondrial fusion protein and failure of which has been implicated in neurodegeneration [38]. *OPA1* oligomerisation also has an important role in maintaining mitochondrial cristae structure, which helps to maintain the stability of the mitochondrial respiratory chain and also prevents the inappropriate release of pro-apoptotic cytochrome C molecules [39].

We sought to characterise the effect of the c.2356-1 G > T variant on mitochondrial function. Given the role of *OPA1* in regulating mitochondrial cristae structure, it is not surprising that *OPA1* variants have been shown to impair mitochondrial bioenergetics in cell and animal models [25, 31, 32]. Patient-derived fibroblasts carrying the c.2356-1 G > T splice site variant had significantly impaired mitochondrial respiration parameters compared with WT controls. Fibroblasts carrying c.2356-1 G > T demonstrated reduced basal, maximal and reserve respiratory capacity consistent with the observation from other cell lines carrying *OPA1* variants [25]. We additionally noted a significant difference between male and female controls lines which may be due to inter-individual variability and/or donor-age effects. Nevertheless, the observed difference between control and patient lines supports a significant bioenergetic deficit due to the c.2356-1 G > T variant. *OPA1* variants also typically impair mitochondrial membrane potential causing further disruption of mitochondrial energetic output and dynamics [40, 41], but how this translates to RGC loss particularly under conditions of heightened physiological stress remains unclear. Artificial stresses such as hypoxia, starvation or glutamate excitotoxicity have been used in vitro to study cell death [39]. Significantly higher levels of cell death were observed in fibroblasts with the c.2356-1 G > T splice site variant following a chemical hypoxic challenge using cobalt chloride, which has been used previously to model hypoxia by stabilising HIF-1α [42]. Hypoxia is a frequently utilised cell stressor as it results in an increase in reactive oxygen species and inhibits mitochondrial respiratory chain activity, which are already impaired by *OPA1* variants [43].

Assessment of mitochondrial networks are a useful marker of mitochondrial function, particularly in the context of *OPA1* variants because of the central role of *OPA1* in regulating mitochondrial dynamics [44]. Mitochondria are highly dynamic organelles, continually undergoing fusion and fission and the state of the mitochondrial network is particularly important in regulating mitochondrial quality control, response to metabolic stress and bioenergetics [45]. Fibroblasts carrying the c.2356-1 G > T variant demonstrated a more fragmented network that had shorter and less numerous branches.

One of the critical challenges in further understanding the pathophysiology of DOA is how different *OPA1* variants contribute to the highly variable phenotype seen in affected patients. Unravelling this complexity is crucial for more accurate genetic counselling and the development of targeted genetic therapies, such as gene augmentation or the use of antisense oligonucleotides [46]. Splice site *OPA1* variants can result in unexpected transcript effects that are not usually studied as part of routine diagnostic testing. In the current study, we used a combination of in silico modelling and in vitro experimental studies on patient-derived fibroblasts to demonstrate that the c.2356-1 G > T splice site variant disrupts the 3’ splice site with activation of an in-frame cryptic acceptor splice site. Given the lack of evidence of significant OPA1 protein degradation in fibroblasts, we speculate that this particular *OPA1* variant could causing the severe multisystemic DOA + phenotype due to a dominant-negative effect. Further evidence to support this putative mechanism could be obtained by direct expression of the mutant p.Cys786_Lys792del protein in an appropriate in vitro cell model already expressing a full complement of the functional WT *OPA1* to demonstrate impaired mitochondrial function due to pathological interference.

## Supplementary information

Nomenclature Statement

## Data Availability

The data contributing to this article is available within the article or its supplementary materials. Otherwise, the data is available on request from the corresponding author.

## References

[1] Yu-Wai-Man P, Griffiths PG, Burke A, Sellar PW, Clarke MP, Gnanaraj L (2010). The prevalence and natural history of dominant optic atrophy due to OPA1 mutations. Ophthalmology.

[2] Yu-Wai-Man P, Griffiths P, Hudson G, Chinnery P (2008). Inherited mitochondrial optic neuropathies. J Med Genet.

[3] Yu-Wai-Man P, Griffiths PG, Gorman GS, Lourenco CM, Wright AF, Auer-Grumbach M (2010). Multi-system neurological disease is common in patients with OPA1 mutations. Brain.

[4] Jurkute N, Majander A, Bowman R, Votruba M, Abbs S, Acheson J (2019). Clinical utility gene card for: inherited optic neuropathies including next-generation sequencing-based approaches. Eur J Hum Genet.

[5] Bonifert T, Karle K, Tonagel F, Batra M, Wilhelm C, Theurer B (2014). Pure and syndromic optic atrophy explained by deep intronic OPA1 mutations and an intralocus modifier. Brain.

[6] Spiegel R, Saada A, Flannery PJ, Burté F, Soiferman D, Khayat M (2016). Fatal infantile mitochondrial encephalomyopathy, hypertrophic cardiomyopathy and optic atrophy associated with a homozygous OPA1 mutation. J Med Genet.

[7] Davies VJ, Hollins AJ, Piechota MJ, Yip W, Davies JR, White KE (2007). Opa1 deficiency in a mouse model of autosomal dominant optic atrophy impairs mitochondrial morphology, optic nerve structure and visual function. Hum Mol Genet.

[8] Alavi MV, Bette S, Schimpf S, Schuettauf F, Schraermeyer HF, Wehrl MVA (2007). A splice site mutation in the murine Opa1 gene features pathology of autosomal dominant optic atrophy. Brain.

[9] Lenaers G, Hamel C, Delettre C, Amati-Bonneau P, Procaccio V, Bonneau D, et al. Dominant optic atrophy. Orphanet J Rare Dis. 2012;7:46. 10.1186/1750-1172-7-46 PMC3526509 22776096

[10] Weisschuh N, Schimpf-Linzenbold S, Mazzola P, Kieninger S, Xiao T, Kellner U (2021). Mutation spectrum of the OPA1 gene in a large cohort of patients with suspected dominant optic atrophy: Identification and classification of 48 novel variants. PLoS One.

[11] Lida K, Ohkuma K, Hayashi T, Katagiri S, Fujita T, Tsunoda K (2016). A novel heterozygous splice site OPA1 mutation causes exon 10 skipping in Japanese patients with dominant optic atrophy. Ophthalmic Genet.

[12] Bolognini R, Gerth-Kahlert C, Abegg M, Bartholdi D, Mathis N, Sturm V (2017). Characterization of two novel intronic OPA1 mutations resulting in aberrant pre-mRNA splicing. BMC Med Genet.

[13] Sun C, Wu X, Bai H-X, Wang C, Liu Z, Yang C, et al. OPA1 haploinsufficiency due to a novel splicing variant resulting in mitochondrial dysfunction without mitochondrial DNA depletion. 2020;42:45–52. 10.1080/13816810.2020.1849313. 10.1080/13816810.2020.1849313 33251885

[14] Yu‐Wai‐Man P, Spyropoulos A, Duncan HJ, Guadagno JV, Chinnery PF (2016). A multiple sclerosis‐like disorder in patients with OPA1 mutations. Ann Clin Transl Neurol.

[15] Yeo G, Burge CB (2004). Maximum entropy modeling of short sequence motifs with applications to RNA splicing signals. J Comput Biol.

[16] Desmet FO, Hamroun D, Lalande M, Collod-Bëroud G, Claustres M, Béroud C (2009). Human Splicing Finder: an online bioinformatics tool to predict splicing signals. Nucleic Acids Res.

[17] Vořechovský I (2006). Aberrant 3’ splice sites in human disease genes: mutation pattern, nucleotide structure and comparison of computational tools that predict their utilization. Nucleic Acids Res.

[18] Brunak S, Engelbrecht J, Knudsen S (1991). Prediction of human mRNA donor and acceptor sites from the DNA sequence. J Mol Biol.

[19] Kelley LA, Mezulis S, Yates CM, Wass MN, Sternberg MJE (2015). The Phyre2 web portal for protein modeling, prediction and analysis. Nat Protoc.

[20] Corporation P. Wizard(R) Genomic DNA Purification Kit Technical Manual #TM050 [Internet]. https://www.promega.co.uk/resources/protocols/technical-manuals/0/wizard-genomic-dna-purification-kit-protocol/.

[21] RNeasy Mini Handbook - (EN) - QIAGEN [Internet]. https://www.qiagen.com/gb/resources/resourcedetail?id=14e7cf6e-521a-4cf7-8cbc-bf9f6fa33e24&lang=en.

[22] Tetro^TM^ cDNA Synthesis Kit [Internet] https://www.bioline.com/tetro-cdna-synthesis-kit.html.

[23] Cox B, Emili A (2006). Tissue subcellular fractionation and protein extraction for use in mass-spectrometry-based proteomics. Nat Protoc.

[24] LDH Assay Kit (Cytotoxicity) (ab65393) | Abcam [Internet]. https://www.abcam.com/ldh-assay-kit-cytotoxicity-ab65393.html.

[25] Sladen PE, Perdigão PRL, Salsbury G, Novoselova T, van der Spuy J, Chapple JP (2021). CRISPR-Cas9 correction of OPA1 c.1334G>A: p.R445H restores mitochondrial homeostasis in dominant optic atrophy patient-derived iPSCs. Mol Ther - Nucleic Acids.

[26] Le Roux B, Lenaers G, Zanlonghi X, Amati-Bonneau P, Chabrun F, Foulonneau T (2019). OPA1: 516 unique variants and 831 patients registered in an updated centralized Variome database. Orphanet J Rare Dis.

[27] Yu-Wai-Man P, Trenell MI, Hollingsworth KG, Griffiths PG, Chinnery PF. OPA1 mutations impair mitochondrial function in both pure and complicated dominant optic atrophy [Internet]. Vol. 134, Brain. Oxford University Press; 2011. p. e164. 10.1093/brain/awq288 PMC3069699 20952381

[28] Amati-Bonneau P, Valentino ML, Reynier P, Gallardo M, Bornstein B, Boissière A (2008). OPA1 mutations induce mitochondrial DNA instability and optic atrophy “plus” phenotypes. Brain.

[29] Barboni P, Savini G, Cascavilla M, Caporali L, Milesi J, Borrelli E, et al. Early macular retinal ganglion cell loss in dominant optic atrophy: genotype-phenotype correlation. Am J Ophthalmol. 2014;158:628–36. 10.1016/j.ajo.2014.05.034 24907432

[30] Anna A, Monika G (2018). Splicing mutations in human genetic disorders: Examples, detection, and confirmation. J Appl Genet.

[31] Kao SH, Yen MY, Wang AG, Yeh YL, Lin AL (2015). Changes in mitochondrial morphology and bioenergetics in human lymphoblastoid cells with four novel OPA1 mutations. Invest Ophthalmol Vis Sci.

[32] Sun S, Erchova I, Sengpiel F, Votruba M (2020). Opa1 Deficiency Leads to Diminished Mitochondrial Bioenergetics With Compensatory Increased Mitochondrial Motility. Invest Ophthalmol Vis Sci.

[33] Weisschuh N, Marino V, Schäferhoff K, Richter P, Park J, Haack TB, et al. Mutations at a split codon in the GTPase-encoding domain of OPA1 cause dominant optic atrophy through different molecular mechanisms. Hum Mol Genet. 2021;31:761–74. 10.1093/hmg/ddab286 PMC8895747 34559197

[34] Brogna S, Wen J (2009). Nonsense-mediated mRNA decay (NMD) mechanisms. Nat Struct Mol Biol.

[35] Li D, Wang J, Jin Z, Zhang Z. Structural and evolutionary characteristics of dynamin-related GTPase OPA1. PeerJ. 2019;7:e7285. 10.7717/peerj.7285 PMC6622160 31328044

[36] Meusser B, Hirsch C, Jarosch E, Sommer T (2005). ERAD: the long road to destruction. Nat Cell Biol.

[37] Alavi MV, Fuhrmann N (2013). Dominant optic atrophy, OPA1, and mitochondrial quality control: Understanding mitochondrial network dynamics [Internet], Molecular Neurodegeneration. BioMed Cent.

[38] Knott AB, Perkins G, Schwarzenbacher R, Bossy-Wetzel E (2008). Mitochondrial fragmentation in neurodegeneration. Nat Rev Neurosci.

[39] Patten DA, Wong J, Khacho M, Soubannier V, Mailloux RJ, Pilon-Larose K (2014). OPA1-dependent cristae modulation is essential for cellular adaptation to metabolic demand. EMBO J.

[40] Song Z, Ghochani M, McCaffery JM, Frey TG, Chan DC (2009). Mitofusins and OPA1 Mediate Sequential Steps in Mitochondrial Membrane Fusion. Mol Biol Cell.

[41] Zhang J, Liu X, Liang X, Lu Y, Zhu L, Fu R (2017). A novel ADOA-associated OPA1 mutation alters the mitochondrial function, membrane potential, ROS production and apoptosis. Sci Rep.

[42] Teti G, Focaroli S, Salvatore V, Mazzotti E, Ingra L, Mazzotti A, et al. The hypoxia-mimetic agent cobalt chloride differently affects human mesenchymal stem cells in their chondrogenic potential. Stem Cells Int. 2018;9. 10.1155/2018/3237253 PMC5872594 29731777

[43] Byrne J, Soh M, Chandhok G, Vijayaraghavan T, Teoh J, Crawford S (2019). Disruption of mitochondrial dynamics affects behaviour and lifespan in Caenorhabditis elegans. Cell Mol Life Sci.

[44] Alavi MV, Fuhrmann N (2013). Dominant optic atrophy, OPA1, and mitochondrial quality control: Understanding mitochondrial network dynamics. Mol Neurodegener.

[45] Hoitzing H, Johnston IG, Jones NS (2015). What is the function of mitochondrial networks? A theoretical assessment of hypotheses and proposal for future research. Bioessays.

[46] Venkatesh A, Zhiyu L, Anne C, Kian Huat L, Jacob K, Robert H, et al. Antisense oligonucleotide mediated increase of OPA1 expression using TANGO technology for the treatment of autosomal dominant optic. Invest. Ophthalmol. Vis. Sci. 2020;61:2755.

