# Supplementary material for: Characterisation of a novel OPA1 splice variant resulting in cryptic splice site activation and mitochondrial dysfunction
Source: Eur J Hum Genet. 2022 May 9;30(7):848–55. doi: 10.1038/s41431-022-01102-0 (PMC9259687; doi:10.1038/s41431-022-01102-0)
Supplement: Supplementary file 1 — Nomenclature Statement [file 41431_2022_1102_MOESM1_ESM.docx]

Genes studied:

OPA1, OPA1 mitochondrial dynamin like GTPase.

ACTB, Actin Beta

GAPDH, Glyceraraldehyde-3-phosphate dehydrogenase

Variants studied:

>OPA1

NM_015560.2:c.2356-1G>T
